# Dynamic disorder is crucial for mitochondrial protein import

**DOI:** 10.1002/pro.70630

**Published:** 2026-05-20

**Authors:** Jakob Schneider, Undina Guillerm, Caroline Simões Pereira, Paul Schanda

**Affiliations:** ^1^ Institute of Science and Technology Austria Klosterneuburg Austria

**Keywords:** avidity, chaperones, import machinery, intrinsic disorder, mitochondria

## Abstract

The import of proteins into mitochondria poses fundamental mechanistic challenges: aggregation‐prone precursor proteins must be maintained in aqueous compartments and threaded through narrow pores without becoming stuck or mislocalized. Recent evidence from mitochondrial protein import studies and other chaperone systems underscores the critical role of dynamics in balancing sufficiently tight binding, promiscuity, specificity, and release. Dynamic binding of client precursor proteins to import machinery components arises naturally from the avidity of their interactions. Conformational entropy enhances their stability, while the multivalent nature of these interactions ensures that client transfer to downstream insertases occurs without a substantial energy barrier. Here, we discuss this emerging paradigm of dynamic protein handling, using examples where dynamic structures have been resolved and highlight outstanding questions.

## 1 REQUIREMENTS OF COMPETENT MITOCHONDRIAL PROTEIN IMPORT

Mitochondria are thought to have originated from the integration of an endosymbiotic α‐proteobacterium into an Asgard archaeal host cell over a billion years ago (Roger et al., 2017). During evolution, mitochondria transferred nearly their entire genome to the nucleus, possibly to circumvent genome damage caused by reactive oxygen species leaking from respiratory chain complexes (Lane, 2018). As a result, more than 99% of mitochondrial proteins are nuclear‐encoded and must be imported and refolded into their final destinations: the outer mitochondrial membrane (OM), intermembrane space (IMS), inner mitochondrial membrane (IM), or matrix. Most mitochondrial proteins are imported post‐translationally after synthesis on cytosolic ribosomes, while a smaller fraction—estimated at 20%—is imported co‐translationally by mitochondria‐associated ribosomes (Luo et al., 2025; Zhu et al., 2025).

**FIGURE 1:**
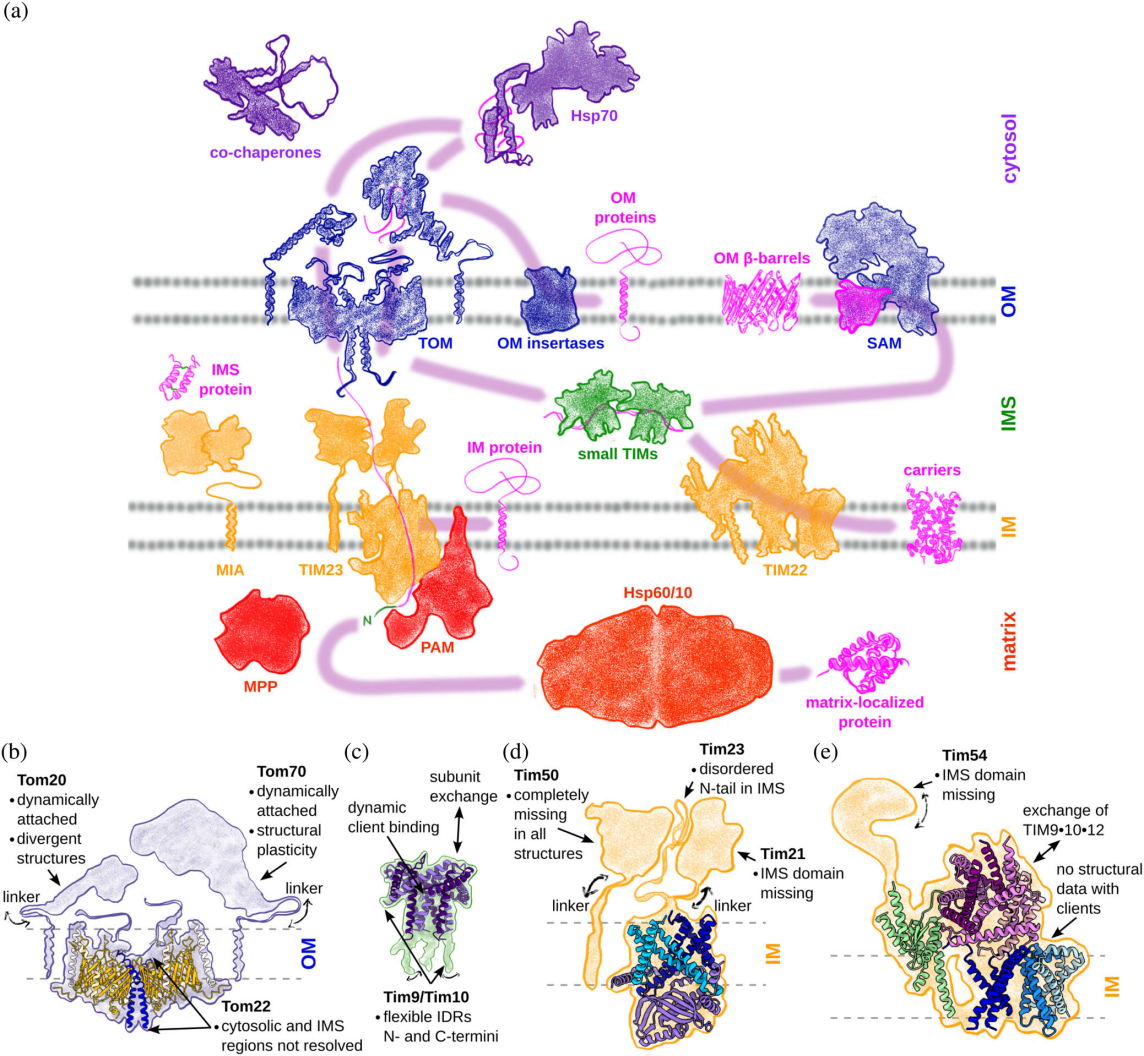
Schematic overview of the mitochondrial import machinery, highlighting the evidence of protein dynamics and disorder. (a) Import components of the cytosol (purple), outer mitochondrial membrane (OM) (blue), intermembrane space (IMS) (green), inner mitochondrial membrane (IM) (yellow) and mitochondrial matrix (red) with their approximate shape. Purple arrows indicate the handover pathways of the precursor proteins (magenta) involved in sorting. (b–e) Representation of individual sorting components with their dynamic elements (Araiso et al., 2019; Baker et al., 2009; Sim et al., 2023; Zhang et al., 2021). (b) TOM complex (Protein Data Bank (PDB): 6JNF). The receptors Tom20 and Tom70 are only loosely attached to the TOM core complex, and show structural flexibility in their folded cytosolic domains. Additionally, Tom20, Tom22, and Tom70 carry flexible linkers and disordered regions in their soluble parts. (c) The IMS chaperones TIM9 ‧ 10 and TIM8 ‧ 13 consist of α‐helical subunits with disordered extensions (PDB: 3DXR). These heterohexameric complexes dynamically exchange individual subunits (Weinhäupl et al., 2021). (d) TIM23 insertase/translocase (PDB: 8E1M). Tim50 is not resolved in available structures and—like Tim21 and Tim23—carries flexible soluble parts. (e) TIM22 insertase (PDB: 6LO8). TIM9 ‧ 10 ‧ 12 exchanges between the TIM22‐bound and free IMS‐located forms, and experiences subunit exchange as in TIM9 ‧ 10 and TIM8 ‧ 13. The IMS domain of Tim54 is missing in available structures.

The import machinery, responsible for the correct sorting of this diverse set of approximately 1000 proteins (Di Bartolomeo et al., 2020; Morgenstern et al., 2017, 2021), is a comparatively small group of specialized proteins (Figure 1a): (i) cytosolic chaperones guide precursor proteins to mitochondria; (ii) receptor domains on the mitochondrial surface recognize these proteins, release them from chaperones, and direct them for translocation through the OM or (for some α‐helical OM proteins) to an insertase; (iii) for certain precursor proteins, IMS chaperones facilitate translocation through the IMS; (iv) β‐barrel proteins are inserted from the IMS into the OM; (v) IM translocases and insertases mediate translocation through the IM or insertion into the IM, with an essential import motor on the matrix side driving this process; (vi) matrix‐targeted proteins undergo further processing and refolding in the matrix. These components were identified by rigorous biochemical and cell biology studies during almost half a century of research. Figure 1a provides a simplified overview. For detailed descriptions of the components and import pathways, the reader is referred to comprehensive reviews on the subject (Bykov et al., 2020; Dimogkioka et al., 2021; Dudek et al., 2013; Endo & Wiedemann, 2025; Genge & Mokranjac, 2022; Hansen & Herrmann, 2019; Jain et al., 2025; Neupert & Herrmann, 2007; Palmer et al., 2021; Pfanner et al., 2025; Wiedemann & Pfanner, 2017; Zarges & Riemer, 2024).

Conceptually, mitochondrial protein import can be viewed as a series of binding events of a precursor protein to consecutive components of the import machinery (chaperones, receptors, translocases, and insertases), each capable of holding the precursor protein, keeping it import competent and preventing aggregation. We refer to this ability of holding a protein as a “holdase function”; this ability is the fundamental function of a chaperone. Reliable sorting from the ribosome to the native environment (OM, IMS, IM, or matrix) needs to meet several functional requirements:

### 1.1 Sufficiently tight binding to prevent aggregation

During import, mitochondrial precursor proteins expose hydrophobic sites that are buried from the solvent after folding/sorting to their native environment. Mitochondrial integral membrane proteins (approximately 350 in yeast; Morgenstern et al., 2021) with highly hydrophobic transmembrane (TM) regions pose the greatest challenge for import. To prevent aggregation, the protein needs to be bound sufficiently tightly to inhibit spontaneous release (discussed below).

### 1.2 Competent handover between sorting components

Efficient transfer of precursor proteins between successive components of the import machinery is as critical as preventing aggregation. For seamless handover, the machinery must specifically recognize downstream sorting components and facilitate transfer. These handover events occur at multiple stages (purple arrows, Figure 1a): (i) transfer between cytosolic chaperones, (ii) transfer to receptor domains at the mitochondrial surface, (iii) release into translocases, (iv) insertion into insertases. In most cases, these transfers proceed without energy consumption, relying instead on the inherent physicochemical properties of the proteins involved. Only a few processes, for example, the adenosine triphosphate (ATP) hydrolysis‐driven conformational changes in Hsp70, couple client binding and release to energy input, enabling transitions between low‐ and high‐affinity states.

### 1.3 Promiscuity of binding

The import machinery must be able to handle about 1000 different proteins of various lengths and physicochemical properties. The import machinery therefore needs to recognize a wide range of sequence features and bind with sufficient affinity.

### 1.4 Specific sorting of clients

The properties of the import machinery components must also convey specificity to mitochondrial precursor proteins. Properties of the nascent polypeptide chain encode the information about its final destination (OM, IMS, IM, and matrix): in about two thirds of all mitochondrial proteins, a variable N‐terminal cleavable amphipathic α‐helix, called presequence, directs proteins to the mitochondrial matrix via the receptor domain of Tom20 (Heller et al., 2026). In other cases, the sorting information is more subtle and encoded in the sequence of the mature protein (termed internal targeting sequences). There is evidence that the hydrophobic character of the α‐helical TM regions of mitochondrial carriers, a class of IM proteins, is specifically recognized by the receptor domain of the OM precursor protein import component Tom70 (Backes et al., 2018). However, Tom70 also handles α‐helical OM proteins, sorting them into the OM for insertion without translocation by the TOM (translocase of the outer membrane) complex. Only subtle property differences control whether Tom70‐bound precursor proteins are inserted into the OM or translocated by TOM. These examples illustrate that, while offering promiscuous client recognition, the mitochondrial import machinery needs to discern mitochondrial precursor proteins from other chaperone‐bound clients in the cytosol and also understand targeting signatures in the precursor protein sequence to prevent missorting.

### 1.5 Transport directionality

The system also requires directionality, where precursor proteins ultimately end in their native compartment. Such directionality is intrinsically provided for precursor proteins that mature after reaching their native environment. Precursor proteins that are sorted by their presequence mature through presequence cleavage by the mitochondrial processing peptidase (MPP) and subsequent folding (Wiedemann & Pfanner, 2017). Many IMS proteins fold after their cysteines are oxidized to form cystine bridges, preventing back‐transfer through TOM (Peleh et al., 2016), and integral OM and IM proteins are retained in the membrane after insertion, given the large energy barrier of extraction.

### 1.6 Import competency of precursor proteins

The translocases and insertases of the import machinery face additional challenges, as membrane pores are limited in size. This essentially excludes the translocation of folded precursor proteins: Tom40, the OM translocase pore, has a diameter of approximately 35 Å (Araiso et al., 2019; Tucker & Park, 2019), allowing at most for translocation of α‐helices devoid of tertiary contacts. Accordingly, the import machinery has to either maintain the precursor proteins in an unfolded and import competent state, or allow their unfolding prior to translocation.

Collectively, the mitochondrial protein import machinery can be understood as a collection of proteins with holdase function. The transfer of precursor proteins along an import pathway has to satisfy the requirements of (i) preventing aggregation, (ii) efficient release, (iii) promiscuity, (iv) specificity, (v) directionality, and (vi) import competency. Some of these requirements appear to be incompatible. We argue that protein dynamics and disorder of mitochondrial precursor proteins as well as the components of the sorting machinery are fundamental features that permit competent import. The next section briefly reviews the basic thermodynamic principles that govern protein binding and folding, which are relevant for the mechanisms of holdase proteins; readers familiar with these general concepts may skip section 2.

## 2 THERMODYNAMIC PRINCIPLES OF PROTEIN–PROTEIN INTERACTIONS

The folded or unfolded states of a protein, the binding of two proteins, and the dynamics of a protein are all fundamentally determined by the differences of the Gibbs free energy (*G*) of the different states of the system. The free‐energy difference between states, ΔG, dictates the equilibrium of the involved states, such as bound and free states in protein–protein interactions:

(1) $$\Delta G_{\text{binding}}=G_{\text{bound}}-G_{\text{unbound}}=\Delta H_{\text{binding}}-T\Delta S_{\text{binding}}.$$

The enthalpy difference (ΔH) reflects changes in favorable/unfavorable interactions, such as hydrogen bonds, charge, dipole, and van der Waals interactions. The entropy difference (ΔS) describes the change of disorder occuring upon binding (including the solvent). The dissociation constant $K_{D}$, that describes the concentration ratio of unbound components and complex, directly depends on $\Delta G_{\text{binding}}$, that is, the more negative $\Delta G_{\text{binding}}$, the higher the population of the complex:

(2) $$K_{D}=\frac{\left[P\right]\left[L\right]}{\left[\mathrm{PL}\right]}=\exp\left(-\frac{\Delta G_{\text{binding}}}{\mathrm{RT}}\right)=\frac{k_{\mathrm{off}}}{k_{\mathrm{on}}}.$$

$K_{D}$ is also related to the kinetic constants of binding ($k_{\mathrm{on}}$) and release ($k_{\mathrm{off}}$).

In most cases, the enthalpic and entropic contributions are antagonistic; for example, upon formation of a salt bridge between two charged sites (negative $\Delta H_{\text{binding}}$ contribution), the interaction restricts these charge‐carrying groups in space, thereby reducing their disorder (negative $\Delta S_{\text{binding}}$ contribution). Likewise, the folding of a protein to a defined three‐dimensional structure dramatically decreases the number of conformations that the polypeptide chain explores. If this loss of entropy is overcompensated by enthalpically favorable interactions and a gain in the solvent entropy resulting from burying hydrophobic parts, then the folded state is thermodynamically favored.

**FIGURE 2:**
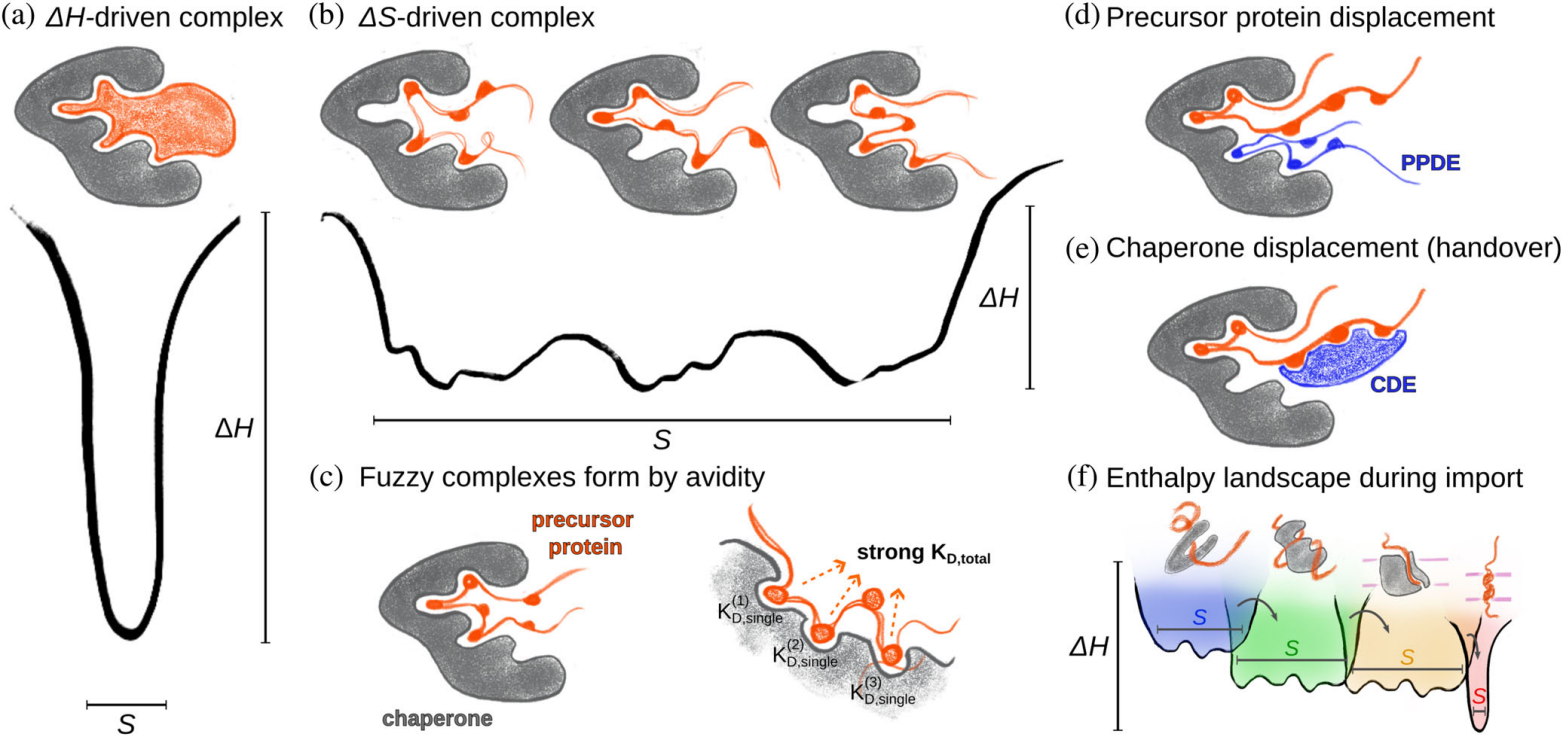
Modes of protein–protein binding and their functional implications. (a) Complex formation driven by enthalpic contributions (e.g., H‐bonds and salt bridges), leading to a well‐defined structure (small entropy). (b) A dynamic, “fuzzy complex” results from a collection of weak interactions in dynamic exchange. The individual interaction partners sample a broad conformational space leading to a large entropy of the complex. (c) Individual weak interactions lead to strong overall protein–protein binding. A high overall affinity results from multivalency (weak local affinity, i.e., high $K_{D,\text{single}}$, but low $K_{D,\text{total}}$). (d) In a “fuzzy complex” the precursor protein can be displaced from a holdase by another dynamic interactor, termed recently a “precursor protein displacement element” (PPDE, Sučec et al., 2026). (e) Similarly to PPDEs, the handover of precursor proteins between sorting components can be understood as displacement of the chaperone by a “chaperone displacement element” (CDE) of the next component. (f) Schematic conformational energy landscapes of a membrane precursor protein during sorting to its final membrane insertion. Transfer between sorting components is facilitated through PPDEs and CDEs and exploits the structural flexibility of the precursor protein. In the process of membrane insertion, the precursor protein assumes a native structure.

Figure 2 schematically depicts protein binding by a conformational energy landscape, where the depth of the wells illustrates the change in *H*, and their width relates to the conformational space accessible to the bound state (*S*). If a protein–protein interaction is driven mostly by favorable intermolecular interactions (H‐bonds, electrostatic contacts), the resulting complex is structurally well‐defined (Figure 2a): The resulting entropy penalty ($\Delta S_{\text{binding}}<0$) is overcompensated by the negative $\Delta H_{\text{binding}}$. Cryo electron microscopy (Cryo‐EM) or crystallography are well suited to resolve such complexes. In contrast, if the bound state explores a variety of conformational states (higher entropy), then a smaller enthalpic contribution suffices to favor complex formation (Figure 2b). Such complexes show an increased amount of disorder and dynamics, and are often referred to as “fuzzy complexes” (Tompa & Fuxreiter, 2008). Rather than featuring a set of strong and well‐defined enthalpic interactions (salt bridges, hydrogen bonds), fuzzy complexes have a large number of redundant, individually weak interactions (multivalency). The low affinity of the individual interactions results in short lifetimes, although in total, the multivalent interaction leads to a high overall complex stability due to avidity and the large entropy contribution of the complex.

Dynamic fuzzy complexes exist through a multitude of binding interactions, which can be seen as individual binding segments connected by linkers (Figure 2c), termed polyvalent binding (avidity) (Mammen et al., 1998). The multiplicity of conformational states, via the associated entropy, contributes to the stability of the complex. The overall binding energy is determined by the sum of the individual $\Delta G_{\text{binding}}$ contributions from each binding site. In a complex, formed by *n* such binding sites, each with a similar individual contribution to binding ($\Delta G^{n}$), the overall dissociation constant is:

(3) $$\begin{array}{rcl} K_{D,\text{total}} & = & \exp\left(-\frac{\Delta G_{1}+\cdots +\Delta G_{n}}{\mathrm{RT}}\right)=\exp\left(-\frac{n\times \Delta G_{\mathrm{avg}}}{\mathrm{RT}}\right)\\ & = & K_{D,1}\cdot \ldots \cdot K_{D,n}=K_{D,\text{single}}^{n}. \end{array}$$

For a case with three possible interaction sites (n=3) with weak individual affinities of $K_{D,\text{single}}=10^{-3}$ M, the avidity results in a complex with a $K_{D,\text{total}}=10^{-9}$ M, that is, very strong binding. While this example is overly simplified (Kane, 2010), it illustrates how multivalency of individually weak interactions leads to complexes with high affinity. In addition to this thermodynamic argument, one can see the high affinity also through kinetic arguments: When one binding site is engaged, the local concentration of the next binding motif is largely increased, effectively increasing $k_{\mathrm{on}}$ of that binding site. To disrupt the complex, the whole array of interactions needs to be released simultaneously, which is an unlikely and therefore rare event (low $k_{\mathrm{off}}$).

The multivalent interaction mode is well suited for a transport system, in which a precursor protein is handed over from one component to the next, as is the case for mitochondrial protein import. The detachment of mitochondrial precursor proteins from one holdase to the next can be a gradual process, where the second component engages with the precursor protein while it is still attached to the previous holdase. This idea of competitive displacement is illustrated in Figure 2d,e, and has been shown to be involved in mitochondrial protein import (see below).

The conformational dynamics in the import of mitochondrial proteins arise from the interaction modes of holdase proteins, which rely on individually weak multivalent interactions. The dynamic nature of the interactions facilitate a transfer via gradual displacement mechanisms, in which the precursor protein is continuously prevented from aggregation. Intrinsically disordered regions (IDRs) may play a role in such interactions: they may act as precursor protein displacement elements (PPDE, Figure 2d) (Sučec et al., 2026), and interaction platforms to transiently bring together several holdases. This might be a reason for the existence of long disordered tails, for example, in TOM and TIM23 (Figure 1).

How strong does the binding of client proteins need to be? While this is difficult to answer generally, here are some general considerations. The affinity needs to be strong enough to prevent client aggregation, but client release must not involve a prohibitively large barrier. The affinities depend on the chaperone and client; more aggregation‐prone clients are presumably more tightly bound than those that are soluble in isolation. Some chaperones have nanomolar *K*_D_ (such as Skp binding highly insoluble membrane proteins, see below). Many others have *K*_D_ values in the low micromolar range, and this weak, transient interaction mode underlies their promiscuous client specificity (Arhar et al., 2021). It is difficult to measure affinities, particularly for aggregation‐prone clients that are not easily amenable to experiments in solution. The affinities relevant for mitochondrial protein import are largely unknown, except for a few cytosolic chaperones (Jores et al., 2018). It is tempting to speculate that there is a gradient of affinities (from cytosolic to mitochondrial components) that drives import into the mitochondrion; however, this assumption remains to be confirmed. Likewise, there is presumably a reduction of the entropy of the precursor protein, from its chaperone‐bound states to the final (folded) state (Figure 2f); direct experimental evidence for this idea is currently lacking, however.

The following sections illustrate these principles in mitochondrial import and other membrane‐protein chaperoning cases. We refer the reader to comprehensive reviews about the general mechanisms of holdases (Bose & Chakrabarti, 2017; Hiller, 2019; Hiller & Burmann, 2018; Sučec et al., 2021).

## 3 DYNAMIC CHAPERONE–CLIENT COMPLEXES

One of the first reports of a chaperone with a full‐length client protein was the study of the bacterial periplasmic chaperone Skp in complex with the outer membrane proteins (OMPs) OmpX and OmpA (Burmann et al., 2013). Skp is a trimeric protein complex of 51 kDa that forms a tentacle‐like structure. It binds OMPs with high affinity (nanomolar *K*_D_; Qu et al., 2007), and guides them across the periplasm. During transport, Skp‐bound OMPs are located in the central hydrophobic cavity between the tentacles, where they behave like an intrinsically disordered protein (IDP), alternating between multiple conformations on sub‐millisecond time scales. Despite this fast local dynamics, the total life time of the complex is on the order of hours. This illustrates the idea outlined in Figure 2 that multiple individually weak interactions can lead to a high overall affinity and long complex life time. When the client protein is too large to be handled by a single Skp trimer, two trimers can come together to form a larger cavity (Schiffrin et al., 2016). Full‐length OmpA comprises a folded soluble domain in addition to the β‐barrel membrane part. When Skp holds OmpA, the membrane‐destined β‐barrel region is located within the cavity as outlined above, while the periplasmic soluble domain is located outside the chaperone and adopts its native folded state (Walton et al., 2009). The highly dynamic binding mode has been found in a number of cases (e.g., Karagöz et al., 2014; Mas et al., 2019), indicating a general mechanism employed by cells to handle unfolded proteins, and it also emerges in mitochondrial protein import, as discussed in the following.

## 4 IMS CHAPERONES INTERACT WITH DYNAMIC DISORDERED PRECURSOR PROTEINS

**FIGURE 3:**
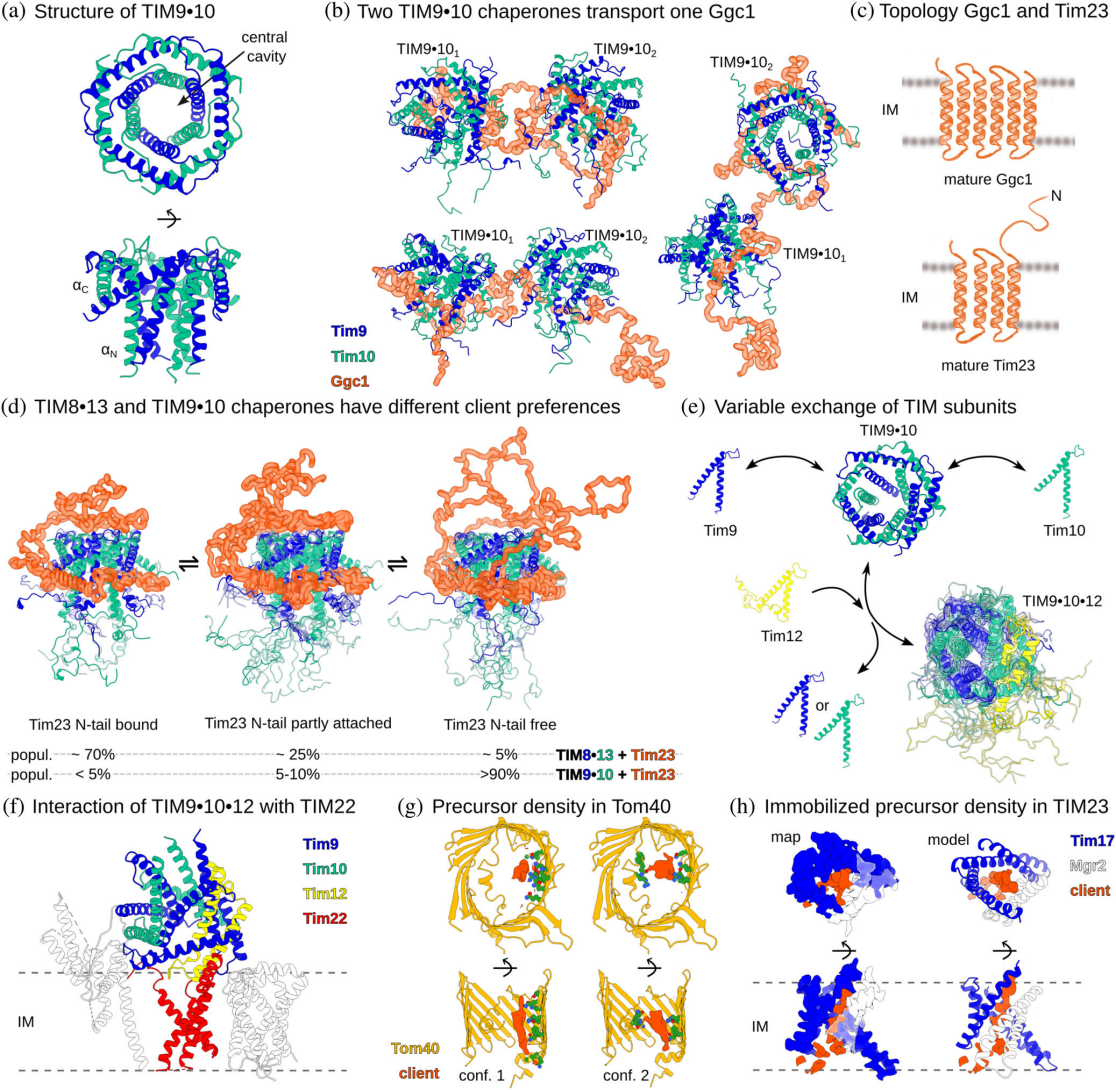
Precursor proteins and their interactions with import proteins. (a) Intermembrane space (IMS) chaperone TIM9‧10 (PDB: 3DXR) (Baker et al., 2009). (b–d) Structures of small TIM chaperones bound to their clients. (b) Two TIM9 ‧ 10 chaperones holding a full‐length mitochondrial carrier (guanosine diphosphate/guanosine triphosphate carrier, Ggc1, shown in orange), as obtained from NMR and small‐angle X‐ray scattering data. Ggc1 is bound to a hydrophobic conserved cleft in multiple conformations, as sketched in Figure 2b (Weinhäupl et al., 2018). (c) Topology of Ggc1 (top) and Tim23 (bottom). (d) Tim23 as a client of small TIM chaperones. Tim23 is held by one small TIM chaperone (several conformers shown). In addition to the hydrophobic interaction of the Tim23 transmembrane (TM) region with the client‐binding cleft of the TIM chaperone, the hydrophilic N‐tail of Tim23 binds to a polar site of the TIM chaperone. Populations of the three ensembles of conformers have been estimated experimentally and are shown below. TIM8 ‧ 13 and TIM9 ‧ 10 differ in their interaction with the N‐tail of Tim23, underlining the differences in specificity (Sučec et al., 2020). (e) Subunits of small TIM chaperones exchange dynamically, co‐existing in equilibrium between complex and free form. Subunit exchange also leads to formation of TIM9 ‧ 10 ‧ 12 (Weinhäupl et al., 2021). (f) Cryo‐EM structure of TIM22, including a TIM9 ‧ 10 ‧ 12 heterohexamer (PDB: 6LO8) (Zhang et al., 2021). (g, h) Cryo‐EM structure of the TOM_cc_ (g, PDB: 9J99) and TIM23 (h, PDB: 9J9B) showing density that points to the presence of precursor protein (Yang et al., 2025). Densities of two out of six identified conformations of the precursor protein in TOM are depicted and interacting Tom40 residues are shown in green. The densities were resolved by three‐dimensional (3D) classification with a local mask on the substrate‐engaged region of a large cryo‐EM dataset and kindly provided by Prof. Long Li. IM, inner mitochondrial membrane.

The mitochondrial IMS hosts a specialized chaperone system composed of the small Tim proteins—Tim8, Tim9, Tim10, Tim12, and Tim13 (Koehler et al., 1998; Sirrenberg et al., 1998; Vial et al., 2002). This TIM chaperone system plays a critical role in facilitating the transfer of some of the most abundant mitochondrial membrane protein precursors from the TOM complex to either the sorting and assembly machinery or TIM22 complexes. The heterohexameric TIM9‧10 and TIM8‧13 chaperones (ca. 70 kDa in size) are formed by either three copies of each Tim9 and Tim10 or Tim8 and Tim13, respectively, arranged in an alternating six‐bladed α‐propeller (Figure 3a). Each subunit consists of two α‐helices (denoted as $\alpha_{N}$ and $\alpha_{C}$ for the N‐ and C‐terminal helices), bound by disulfide bridges at conserved sites. The extreme N‐ and C‐termini are highly flexible, as quantified by nuclear magnetic resonance (NMR) (Weinhäupl et al., 2018). Based on x‐ray structures of these chaperones (Baker et al., 2009; Beverly et al., 2008; Webb et al., 2006), several speculative models of the binding mode with mitochondrial carrier precursor proteins have been proposed (Beverly et al., 2008; Vergnolle et al., 2005; Webb et al., 2006). The fundamental difference between these models—and the fact that they all turned out incorrect—illustrates that static structures of the mitochondrial import machinery without bound precursor proteins are not sufficient to understand the molecular mechanisms of binding and transfer.

An integrated biophysical and cellular study, based on NMR, small‐angle x‐ray scattering and mutagenesis data in yeast, has clarified how full‐length mitochondrial carrier proteins bind to the TIM9 ‧ 10 chaperone (Weinhäupl et al., 2018; Figure 3b). The precursor protein is wrapped around the chaperone in a highly dynamic manner. NMR experiments identified a region between the $\alpha_{N}$ and $\alpha_{C}$ helices of TIM9 ‧ 10 as the primary location to which precursor proteins bind; this part of the chaperone comprises a patch of conserved hydrophobic residues. The individual interactions between the precursor protein and the chaperone are continuously formed and broken resulting in a multitude of locally bound and unbound states. NMR dynamics experiments revealed that these dynamics occur on a time scale of a few milliseconds. However, the overall life time of the complex is much longer: the time required to transfer a mitochondrial carrier precursor protein from one TIM9 ‧ 10 complex to another was shown to be several hours (Weinhäupl et al., 2018). While this observation is not a direct measure of the affinity—such measurements are impeded by the low solubility of the clients—it suggests that the dissociation constant is much smaller than 1 μM: micromolar *K*_D_ values usually are related to off‐rates ($k_{\mathrm{off}}$) in the time scale of μs to ms, orders of magnitude faster than what these client‐transfer experiments revealed.

Although the complex is formed through a multitude of interactions and stabilized by avidity, single point mutations that replace a hydrophobic residue with a polar one cause severe growth and import defects and abolish precursor binding by TIM9‧10 (Weinhäupl et al., 2018). These findings show that these chaperone–client complexes are optimized for weak binding with only a shallow $\Delta G_{\text{binding}}$; we speculate that more tight binding may be disrupting the transfer function of the chaperones by hindering client release downstream.

A remarkable feature of the small TIM chaperone–client complexes is the varying stoichiometry of client and chaperone. This resembles the mechanism of bacterial Skp discussed above, indicating a general feature found in precursor protein transport systems to guarantee efficient transport of diverse clients. Full‐length mitochondrial carriers (ca. 35 kDa integral IM proteins with six TM α‐helices, Figure 3c), such as the adenosine diphosphate/ATP carrier (Aac3) or GDP/GTP carrier (Ggc1) recruit two hexameric TIM9 ‧ 10 chaperones (Figure 3b), while the shorter Tim23 client protein (four TM α‐helices, Figure 3c,d) recruits only one TIM chaperone (Sučec et al., 2020; Weinhäupl et al., 2018).

The small TIM chaperones also provide lessons about client specificity (Sučec et al., 2020). While the two IMS chaperones in yeast—TIM9‧10 and TIM8‧13—have the same architecture, they show different preferences for substrate proteins: TIM9‧10 has a 10‐fold higher affinity to the mitochondrial carrier Ggc1 than to Tim23. In contrast, the homologous TIM8‧13 slightly favors binding to Tim23 over Ggc1. What is the reason for this preference? It is related to a ca. 100 residues long hydrophilic N‐terminal IMS extension (N‐tail) in Tim23 (in addition to its TM α‐helices). Structural and dynamical analyses have revealed that this additional hydrophilic tail forms additional contacts with the chaperones, and that these differ between TIM9‧10 and TIM8‧13 (Figure 3d): Tim23's hydrophilic tail forms dynamic contacts with a hydrophilic patch on TIM8‧13, but much less with TIM9‧10 (Figure 3d). It is noteworthy that all these interactions are dynamic, and that even such dynamic contacts can contribute significantly to the selectivity and stability of the complex.

The hexameric small TIM chaperones also feature subunit exchange dynamics: the hexamer coexists with free subunits. Subunits enter and exit the hexamer on a time scale of tens of minutes at 25°C (Weinhäupl et al., 2021). This subunit exchange even occurs when a full‐length precursor protein is wrapped around the chaperone. This observation supports the notion of a highly dynamic hexameric chaperone assembly, implying that the presence of the precursor protein does not restrict subunit exchange.

A key question is how mitochondrial carriers are ultimately inserted into the IM by the TIM22 insertase. Cryo‐EM structures of the TIM22 insertase (Qi et al., 2021; Zhang et al., 2021) have provided important insights (Figure 3f). The TIM9‧10‧12 chaperone assembly, which resembles the TIM9‧10 chaperone, except that one subunit is replaced by a Tim12 subunit, is tightly associated to TIM22. Thus, TIM22 has an IMS chaperone integrated. Structural studies of TIM22 with precursor proteins are missing, and the transfer mechanism remains speculative. One possibility is that full‐length mitochondrial carrier precursor proteins “slide” over from TIM9‧10 (during the IMS transfer) to a TIM22‐anchored TIM9‧10‧12. Alternatively, the TIM9‧10 complex that transports the precursor protein may exchange one of its subunits for a Tim12 (as shown above, this can happen spontaneously), and this precursor protein‐loaded TIM9‧10‧12 the gets recruited into the TIM22 complex for subsequent membrane insertion of the precursor protein.

## 5 PRECURSOR PROTEINS ENGAGED IN TRANSLOCASES AND INSERTASES

Besides the above‐described complexes of mitochondrial carriers and IMS chaperones, recent studies provided structural information about the engagement of precursor proteins with translocases and insertases of the import machinery. In particular, cryo‐EM structures revealed additional densities that were ascribed to precursor proteins engaged in these membrane proteins (Agip et al., 2025; Yang et al., 2025). Agip et al. observed additional density in the lumen of the Tom40 barrel (Agip et al., 2025). This density was interpreted to correspond to the presequence of aldehyde dehydrogenase (pALDH), a common model presequence‐dependent protein import, that was incubated with the studied TOM complex prior to the plunge‐freezing of the cryo‐EM grids. Interestingly, the authors reported three fairly distinct locations of this density within the barrel, which suggests that precursor proteins can adopt several distinct conformations during translocation. The presence of distinct pathways through the Tom40 translocase was suggested previously by cross‐linking mass spectrometry data (Shiota et al., 2015). In that work it was proposed that different types of precursor proteins would employ distinct pathways through Tom40, assisting sorting. However, the cryo‐EM data by Agip et al. suggests that the same precursor protein dynamically samples different pathways, thereby mirroring the highly dynamic binding of mitochondrial carriers to the TIM chaperones (see above).

In another approach, the TOM‐TIM23 supercomplex was arrested with an artificial precursor protein that carries a stable folded domain on both the cytosolic side of TOM and the matrix side of TIM23 (Yang et al., 2025; Zhou et al., 2023). Consequently, the TOM and TIM23 are tethered together with this long precursor protein mimic. Akin to the behavior of pALDH in the structure of TOM, the authors reported several distinct densities inside the lumen of Tom40 (Figure 3g). The low resolution of this density—possibly the result of dynamic disorder—did not allow the atomic‐level modeling of the precursor protein or description of secondary structure features. In TIM23, the density of the precursor protein was sufficient to interpret it as α‐helical (Figure 3h). Possibly, the TIM23 translocase, which is narrower than the Tom40 lumen, restricts the secondary structure of the precursor protein.

Collectively, precursor proteins engaged in translocases and insertases appear to undergo significant dynamics, although this interpretation is inferred only from single‐particle analysis cryo‐EM, that is, of frozen particles. Other techniques, such as single‐molecule techniques or NMR may shed more light on the dynamic properties.

## 6 THE DYNAMIC HANDLING OF PRECURSOR PROTEINS BY TOM RECEPTORS

**FIGURE 4:**
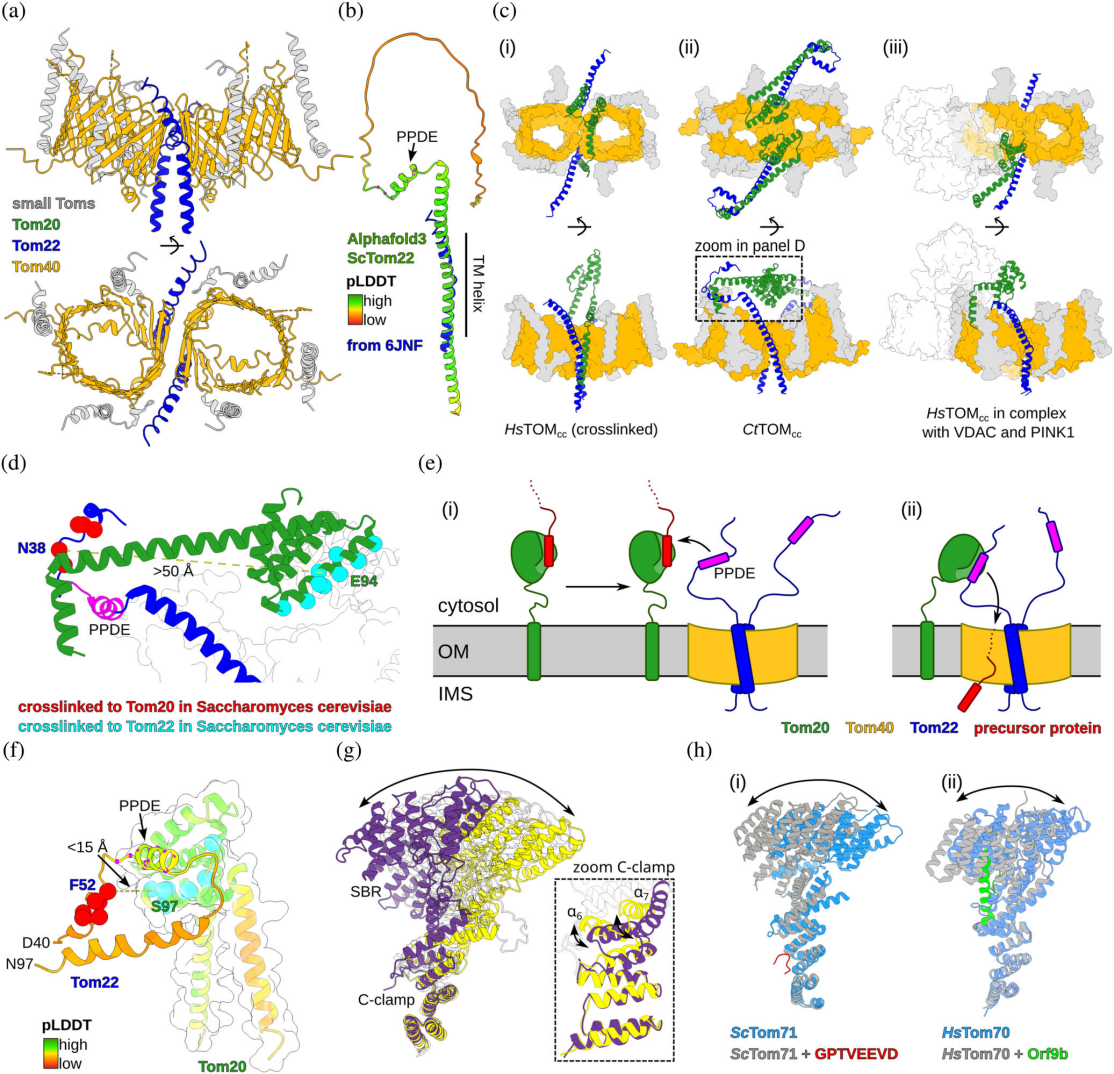
Structural heterogeneity of the TOM complex. (a) Cryo‐EM structure of the TOM core complex (TOM_cc_, PDB: 6JNF) (Araiso et al., 2019). In the model, residues 1–86 (cytosolic) and 136–152 (intermembrane space [IMS]) of Tom22 are not resolved, indicating flexbility. Tom20 and Tom70 were not observed in this structure. (b) AlphaFold3 model of *Sc*Tom22 colored by predicted local distance difference test score (Abramson et al., 2024). The part resolved in (a) is shown in blue. $C_{\alpha }$ atoms of PPDE residues highlighted in magenta. (c) Recent TOM_cc_ structures resolved density for Tom20 and Tom22, showing different orientations (PDBs: (i) 8XVA; (ii) 9I7S; (iii) 9EII) (Agip et al., 2025; Callegari et al., 2025; Su & Wang, 2024). (d) Zoom onto the structural model by Agip et al., showing the Tom20‐Tom22 contact. Residues analogous to cross‐linking experiments in *Saccharomyces cerevisiae* (Shiota et al., 2011) are shown as spheres (cyan Tom20, red Tom22). In this model, they are separated by 30–50 Å. (e) Recently proposed model of the Tom22 function, where a short α‐helical motif in its cytosolic part serves as a precursor protein displacement element (PPDE) to release Tom20‐ or Tom70‐bound precursor proteins (Sučec et al., 2026). (f) PPDE‐mediated *Sc*Tom22‐*Sc*Tom20 interaction, modeled by AlphaFold3 (Abramson et al., 2024). This interaction mode brings the cross‐linked residues in close neighborhood (compare [d], same residues highlighted). (g) Two microsecond molecular dynamics simulation of *Sc*Tom70_cyt_, performed for this review. Ten snapshots are shown from this trajectory, overlayed at the C‐clamp (bottom). The two extreme structures of the observed dynamics are shown in yellow and purple. (h) Experimental evidence for structural plasticity in Tom70 and Tom71, aligned at the C‐clamp (Bachochin et al., 2026; Gao et al., 2021; Li et al., 2009). Motion of (i) free and bound *Sc*Tom71 with a peptide corresponding to the Hsp70 C‐terminus (PDBs: 3FP3, 3FP4) and (ii) free and bound *Hs*Tom70 with the viral Or9b protein (PDBs: 9PKQ, 7DHG). OM, outer mitochondrial membrane.

Within the last decade, the structural characterization of the TOM complex, the central import gate of mitochondria, has made enormous progress, with a multitude of cryo‐EM structures from several organisms from human to yeast (Agip et al., 2025; Araiso et al., 2019; Bausewein et al., 2017; Callegari et al., 2025; Guan et al., 2021; Model et al., 2008; Ornelas et al., 2023; Su et al., 2022; Su & Wang, 2024; Tucker & Park, 2019; Wang et al., 2020; Yang et al., 2025; Zhou et al., 2023). The architecture of the TOM core complex (TOM_cc_) is largely conserved in all available structures, consisting of two Tom40 pores and two copies of Tom22, whose TM helices are located centrally between the pores. Conserved lipids and the small Tom subunits (Tom5, Tom6, and Tom7) surround the Tom40 barrel (Figure 4a) (Agip et al., 2025; Araiso et al., 2019; Ornelas et al., 2023; Tucker & Park, 2019; Wang et al., 2020). The TOM_cc_ functions together with the so‐called receptor proteins Tom70 and Tom20, which recruit precursor proteins for translocation. Of note, Tom70 is missing in all structures of the TOM_cc_ known to date. Similarly, Tom20 is missing in most structures. The absence of the receptor proteins Tom70 and Tom20 in most structures is presumably related to the fact that these proteins, inserted into the OM with a single TM helix, can diffuse freely in the membrane and associate only transiently to TOM_cc_. This diffusion has been directly shown for Tom20 (Bhagawati et al., 2021; Sučec et al., 2026). Likewise, the cytosolic part of Tom22, often referred to as the “central receptor” is missing in most structures (Figure 4a,b). In the few structures in which the cytosolic parts of Tom20 and Tom22 have been observed at least partly, these appear contradictory: structures of human TOM_cc_ obtained with chemical cross‐linkers (Su et al., 2022; Su & Wang, 2024) show Tom20 anchored to the TOM_cc_ by its TM helix while the cytosolic domain is located at the cytosolic opening of the Tom40 pore (Figure 4c, i) (Su & Wang, 2024). In contrast, structures from *Neurospora crassa* (Ornelas et al., 2023) and *Chaetomium thermophilum* (Agip et al., 2025) TOM_cc_ in micelles showed Tom20 in a different conformation with its cytosolic domain close to the Tom40 pores and interacting with a functionally relevant stretch of Tom22 (44–57, see below) close to the TM helix of Tom20 (Figure 4c, ii). In all these structures, the features of TOM_cc_ were well‐resolved, while the Tom20 subunits were less defined. 3D classification of particles in the same data set also showed that the Tom20 receptor adopts diverse states (Agip et al., 2025). Interestingly, the cryo‐EM structural models not only diverge from each other, but they also disagree with in vivo cross‐linking data (Figure 4d) (Shiota et al., 2011), suggesting that the cryo‐EM data do not reflect the in vivo conformations entirely.

The variability of the Tom20 and Tom22 conformations in the available cryo‐EM structures suggests flexibility. Recent results reveal a functional importance of this flexibility and point to a fundamental role of Tom22, unrelated to direct precursor protein binding (Figure 4e) (Sučec et al., 2026): the cytosolic part of Tom22 was shown to be intrinsically disordered, and only a small part forms a transient helix (55–68 in *Saccharomyces cerevisiae*, 44–57 in *Chaetomium thermophilum*), at an estimated population of about 30%. Disorder in the cytosolic region of Tom22 had also been reported in *Arabidopsis thaliana* (Rimmer et al., 2011), indicating that a disordered segment in Tom22 is a conserved feature. Rather than acting as a folded receptor domain, Tom22 is proposed to offer a “precursor protein displacement element” (PPDE, Figure 2d) for precursor protein release at the location of the translocation pore Tom40. The transient helix of Tom22 can bind Tom20 and Tom70 at their respective client binding sites, and therefore outcompete precursor proteins (Figure 4e). This binding mode of Tom20 and Tom22 is in agreement with cross‐linking data (Figure 4f). This proposed model (which involves dynamic multivalent binding) provides an elegant mechanism of precursor protein translocation: the receptor proteins Tom20 and Tom70 may capture clients, possibly while not bound to TOM, and once these client‐loaded receptors are above the TOM pore, Tom22 drives the client release into the translocation pore.

## 7 THE RECEPTOR AND CHAPERONE HUB TOM70 EXISTS IN MULTIPLE CONFORMATIONS

There is strong evidence that the “holdase” components of the import machinery have structural flexibility, that is, that their structure depends on the presence of interaction partners. A particularly interesting example is the import receptor Tom70, which is N‐terminally anchored to the OM with a single TM helix, and has a ca. 60 kDa large folded domain (Tom70_cyt_) composed of 11 α‐helical tetratricopeptide repeat (TPR) motifs, separated from the TM α‐helix with a long IDR. Tom70 serves as the primary receptor for proteins bearing internal, non‐cleavable targeting signals (Backes et al., 2018, 2021). Initially recognized for its role in importing IM α‐helical proteins such as mitochondrial carriers (Young et al., 2003), Tom70 has since been shown to handle a broader range of hydrophobic, aggregation‐prone polypeptides (Backes et al., 2021). These include β‐barrel OM proteins, which traverse the OM via the TOM complex before insertion from the IMS, as well as α‐helical OM proteins that are directly inserted from the cytosol by specialized insertases: Mitochondrial import protein in yeast (Becker et al., 2011; Popov‐Čeleketić et al., 2008; Waizenegger et al., 2005), pATOM36 in trypanosomes (Vitali et al., 2018), and MTCH2 in humans (Guna et al., 2022). These divergent pathways controlled by the same receptor protein lead to currently unresolved mechanistic questions: Which properties determine sorting through the TOM complex or handover to an insertase? How does Tom70 contribute to this sorting? Does Tom70 offer separate client binding sites for differently targeted clients or does it interact with them through different dynamic modes?

Tom70 not only binds precursor proteins, but also functions as a central interaction hub for cytosolic chaperones, co‐chaperones (like J‐domain proteins; Opaliński et al., 2018; Papić et al., 2013) and precursor proteins. The structure of Tom70_cyt_ shows two distinct interaction surfaces. The first three TPRs form a conserved basic binding clamp (referred to as chaperone clamp, C‐clamp), responsible for recruitment of conserved acidic stretches (EEVD motif) in cytosolic chaperones such as Hsp70 and Hsp90 (Young et al., 2003). The other TPRs from a deep hydrophobic groove, able to interact with client proteins (substrate binding region, SBR). These two independent interaction surfaces are linked by two long α‐helices central to the folded domain (denoted as $\alpha_{7}$ and $\alpha_{25}$). It is still unclear, how a chaperone‐bound precursor protein is transferred to Tom70. In this context, it is assumed that there is an allosteric coupling between the C‐clamp and the SBR (Bachochin et al., 2026; Li et al., 2009, 2010), such that chaperone binding would enhance the affinity for precursor protein binding (Ayinde et al., 2022; Bachochin et al., 2026; Gao et al., 2021; Gordon et al., 2020). As allostery is intimately linked to the dynamic conformational ensembles (Motlagh et al., 2014), the motions of Tom70 have been studied intensely.

Already in its unbound state Tom70_cyt_ features long‐range conformational dynamics. This is illustrated by molecular dynamics (MD) simulations, which we performed with *S. cerevisiae* Tom70_cyt_. Figure 4g shows an overlay of snapshots from such a simulation (0.4 μs steps, 2 μs total simulated time), highlighting the large‐amplitude motion of the two ligand binding surfaces relative to the other. Similar MD simulations for human Tom70_cyt_ have been reported by others (Bachochin et al., 2026). Experimental structures of *Sc*Tom70_cyt_ (Wu & Sha, 2006), *Hs*Tom70_cyt_ (Bachochin et al., 2026; Gao et al., 2021; Gordon et al., 2020; Sherer et al., 2025) and the yeast Tom70 homolog *Sc*Tom71cyt (Li et al., 2009) capture some of this structural diversity. In a recent crystallographic study of *Hs*Tom70_cyt_ (Bachochin et al., 2026), the asymmetric unit cell showed two different states, induced by contacts between neighboring Tom70_cyt_ molecules in the crystal, referred to as “open” and “closed” states. Structures of *Hs*Tom70_cyt_ bound to the severe acute respiratory syndrome coronavirus 2 (SARS‐CoV2) protein Orf9b (Gao et al., 2021; Gordon et al., 2020), together with the unbound *Hs*Tom70_cyt_ (Bachochin et al., 2026), also revealed domain motion. Evidence of domain reorientation was found in the presence of peptides corresponding to the acidic C‐terminal stretch of Hsp70 and Hsp90 to the C‐clamp of *Sc*Tom71_cyt_ (Li et al., 2009, 2010). While the SBR of Tom70 has been identified for over two decades (Li et al., 2009; Wu & Sha, 2006), no structures of Tom70_cyt_ bound to precursor proteins have been reported to date. This is likely caused by a dynamic interaction mode. A recent NMR study provided direct structural data of precursor protein fragments bound to the SBR of Tom70_cyt_ (TM fragments of mitochondrial carriers, and of the voltage‐dependent anion channel) (Sučec et al., 2026). Besides showing that these precursor proteins bind into the hydrophobic SBR, this study has also revealed that *Sc*Tom70_cyt_ exists in two stable conformations, and that client binding alters the equilibrium of these states. Whether the states observed herein are related to the states reported by crystallography remains to be clarified. It is likely that other components with holdase function have similar structural diversity, and that the exchange between structural states is related to distinct functions.

## 8 OPEN QUESTIONS

Recent cryo‐EM structures have provided highly valuable static snapshots of the import machinery but several critical questions remain unanswered: What drives mitochondrial protein import? Most of the discussed transfer steps are reversible. Until a precursor protein passes the strict TIM23 translocase or is inserted into membranes, it can be handed over back and forth in the transport chain. For this reason, it is intriguing to study if the cell uses an affinity gradient for successive sorting components to force directionality.

How are precursor proteins handed over? We propose that transfer of a precursor protein may involve an active engagement of the downstream component, along the lines of what we have described as precursor protein displacement mechanism or chaperone displacement mechanism in Figure 2d,e, which seems to be the function of Tom22. If this handover fails, what are the molecular consequences? Methods well equipped to study dynamic systems may help understand the mechanisms that take care of these processes.

How do the components of the TOM complex cooperate to enable translocation through the membrane? The dynamic interaction between cytosolic chaperones, the Tom20 and Tom70 receptors (which are mobile in the OM), and the TOM_cc_ still remains incompletely understood. An important question is related to the mechanism by which precursor proteins are transferred from the chaperones to the receptors (e.g., is there an affinity gradient involved?), and then from the receptors to the downstream components, namely the Tom pore for translocation or the OM insertase. Tom70 still poses a number of questions. What is the function of the different states observed in NMR, crystallography, and cryo‐EM? How does Tom70 determine the fate of a recruited precursor protein? Which other proteins regulate its function? Additionally, the role of Tom22 is also not fully understood. How is the PPDE function related to chaperone recruitment? The cytosolic IDR could function as a multivalent interaction hub for a plethora of different interaction partners. It is important to understand the role of Tom40 in precursor protein sorting. Does Tom40 contain multiple distinct import pathways? Are both pores active simultaneously?

Finally, while our focus here has been on successful import, we are only beginning to understand the mechanisms at play when import fails. These processes may involve back‐transfer, degradation, or storage (e.g., in MitoStores; Krämer et al., 2023), followed by resolubilization. We argue that investigating the flexibility of the components and precursor proteins involved will be a key step toward unraveling these processes.

Addressing these questions is challenging, as many precursor proteins are inherently unstable or insoluble, complicating the application of biophysical techniques. It is likely that progress in this field will involve multiple techniques, such as time‐resolved cryo‐EM, single‐molecule spectroscopies, NMR, and biochemical techniques.

## 9 CONCLUSION

The efficient, specific, and yet promiscuous transfer of precursor proteins between components of the mitochondrial import machinery is essential for successful protein import. Dynamic disorder emerges as a central feature, enabling the system to balance the various, seemingly contradictory requirements for import components: sufficiently tight binding, efficient release, promiscuity, specificity, directionality while enabling import competency. This dynamic behavior arises from multivalent binding: the fuzzy interaction also enables the displacement of one bound protein by another (see Figures 2d and 4e). We propose that this mechanism may be more widespread, with disordered regions in other translocases and insertases potentially fulfilling similar roles, and that a similar gradually dynamic displacement is involved in client handover between import components (Figure 2e).

Major advances in the field have been obtained over the last decade from static high‐resolution crystallography and cryo‐EM structures. We believe that the next advancements must include the explicit characterization of the fundamental protein dynamics and contribution of disordered regions, which shall clarify mechanisms of handover, directionality, and precursor protein maturation.

## AUTHOR CONTRIBUTIONS

**Jakob Schneider:** Conceptualization; writing – original draft; writing – review and editing. **Undina Guillerm:** Conceptualization; writing – review and editing; resources. **Caroline Simões Pereira:** Resources; writing – review and editing. **Paul Schanda:** Conceptualization; funding acquisition; writing – original draft; writing – review and editing; supervision.

## CONFLICT OF INTEREST STATEMENT

The authors declare no potential conflict of interest.

## Data Availability

Data sharing not applicable to this article as no datasets were generated or analysed during the current study.
